# Psychometric Properties of a Developed Questionnaire to Assess Knowledge, Attitude and Practice Regarding Vitamin D (D-KAP-38)

**DOI:** 10.3390/nu9050471

**Published:** 2017-05-08

**Authors:** Parisa Amiri, Golaleh Asghari, Hoda Sadrosadat, Mehrdad Karimi, Atieh Amouzegar, Parvin Mirmiran, Fereidoun Azizi

**Affiliations:** 1 Research Center for Social Determinants of Endocrine Health & Obesity Research Center, Research Institute for Endocrine Sciences, Shahid Beheshti University of Medical Sciences, Tehran 1985717413, Iran; amiri@endocrine.ac.ir (P.A.); hoda.sadrosadat@gmail.com (H.S.); mehrdadstat@gmail.com (M.K.); 2 Nutrition and Endocrine Research Center, Research Institute for Endocrine Sciences, Shahid Beheshti University of Medical Sciences, Tehran 1985717413, Iran; g_asghari@hotmail.com (G.A.); mirmiran@endocrine.ac.ir (P.M.); 3 Department of Epidemiology and Biostatistics, School of Public Health, Tehran University of Medical Sciences, Tehran 1985717413, Iran; 4 Endocrine Research Center, Research Institute for Endocrine Sciences, Shahid Beheshti University of Medical Sciences, Tehran 1985717413, Iran; azizi@endocrine.ac.ir

**Keywords:** vitamin D, knowledge, attitude, practice, validity, reliability, D-KAP-38

## Abstract

The aim of this study was to develop a valid and reliable questionnaire to assess vitamin D-related knowledge, attitude and practices in Tehranian adults, who may be at increased risk of vitamin D deficiency. This study was conducted on 572 individuals, aged ≥20 years from public health care centers in Tehran, Iran. Based on results of a literature review and in-depth interviews, the 38-item vitamin D-related KAP questionnaire (D-KAP-38) with four subscales was developed: (1) general knowledge; (2) nutritional knowledge; (3) attitudes; (4) behaviors. Validity of the D-KAP-38 questionnaire was assessed, utilizing face, content, and construct validity methods. Internal consistency was calculated to assess reliability of the current developed questionnaire. A total of 572 (54.1% female) adults, aged 30.2 ± 7.9 years, participated in the study. All items were perceived as relevant and comprehendible by participants. Content validity was confirmed by a panel of experts. The internal consistency, as measured by Cronbach’s alpha coefficients, exceeded the minimum reliability standard of 0.60 for four subscales. Exploratory factor analysis suggested a four-factor construct and the results of the confirmatory factor analysis indicated acceptable fit indices for the proposed model. No ceiling effects were observed except for general knowledge (1.2%). Floor effects detected were 0%, 1.1%, 2.4%, and 8.7% for practice, attitude, general knowledge, and nutrition knowledge, respectively. General knowledge had the highest score (79.59 ± 14.52) and nutrition knowledge had the lowest (42.58 ± 20.40), among the four sub-scales. Results confirm the initial validity and reliability of D-KAP-38 questionnaire. Further investigations in different populations are recommended.

## 1. Introduction

Besides the central role of vitamin D in mineral metabolism, data reveals that low levels of this important micronutrient might be associated with risk of various cancers, cardiovascular disease, diabetes, autoimmune disorders, infection, chronic kidney disease, and muscle metabolism [1,2,3]. Worldwide, a considerable prevalence of vitamin D deficiency (25-hydroxy vitamin D < 20 ng/mL) has been reported among different ethnicities and age groups [4,5]. In Iran several studies from different urban areas have shown a high prevalence of vitamin D deficiency in different sub groups, including women, adolescents and taxi drivers [6,7,8].

Although vitamin D deficiency is a multi-factorial phenomena that is influenced by several socio-environmental factors, it is evident that individuals’ knowledge, attitude, and practice are associated with this condition [9,10]. In this regard, despite sufficient evidence on the various determinants of vitamin D deficiency including lack of sun exposure and dietary intake, studies conducted on general practitioners, university and school students, general adult populations, and athletes mostly found participants to have inadequate knowledge regarding the role of sunlight in vitamin D production, the function and sources of vitamin D, and also negative attitudes and behaviors toward sunlight [11,12,13,14,15,16,17,18,19,20,21]. Hence, it seems understanding individuals’ knowledge, attitude and practice (KAP) regarding vitamin D is essential in developing related health promotion programs.

Limited instruments have been so far developed to assess individuals’ vitamin D-related KAP. In some previous studies conducted on vitamin D, KAP was evaluated using a number of questions rather than using a structured questionnaire [11,12,17,18,20]. On the other hand, the psychometric properties of structured questionnaires used in some other studies were mainly assessed by face and content validity and none of them utilized statistical approaches to examine construct validity of the applied instruments. While vitamin D-related behaviors are rooted in culture, social norms, religion, personal expectations and environmental reinforcements, most existing questionnaires have been developed in Western communities [11,14,17,20] with high levels of socio-environmental differences with Eastern countries, in particular with Muslim populations. To fill the gap in the current literature, this study is one of the first efforts aimed at developing a valid and reliable questionnaire to assess vitamin D-related KAP in Tehranian adults who are at increased risk of vitamin D deficiency.

## 2. Materials and Methods

### 2.1. Participants and Data Collection

Participants were adults aged ≥20 years, selected and recruited from public health care centers in Tehran. To recruit a representative adult population for the current study, the city of Tehran was divided into the North and South areas, corresponding to high and low socio-demographic status, respectively. From among all districts, located in each of the socio-demographic areas, one district of Tehran metropolitan city was randomly selected, following which three public health centers were randomly selected from each district, resulting in 572 participants from 6 health centers. The ethics committee of the Research Institute for Endocrine Sciences (RIES), Shahid Beheshti University of Medical Sciences approved the study (ethical approval code: 22ECRIES91/12/08). All participants provided written informed consent before the interviews and explicit permission was sought for audio taping.

### 2.2. Measures

The final version of the vitamin D-related knowledge, attitude, and practice questionnaire included 38 items (D-KAP-38, Supplementary Materials), developed through literature review and in-depth interviews. The demographic questionnaire included information on age, gender, education, parity, occupation, marital status, and residential area, as well as on vitamin D supplement consumption history.

### 2.3. Scale Development Procedure

**Figure 1:**
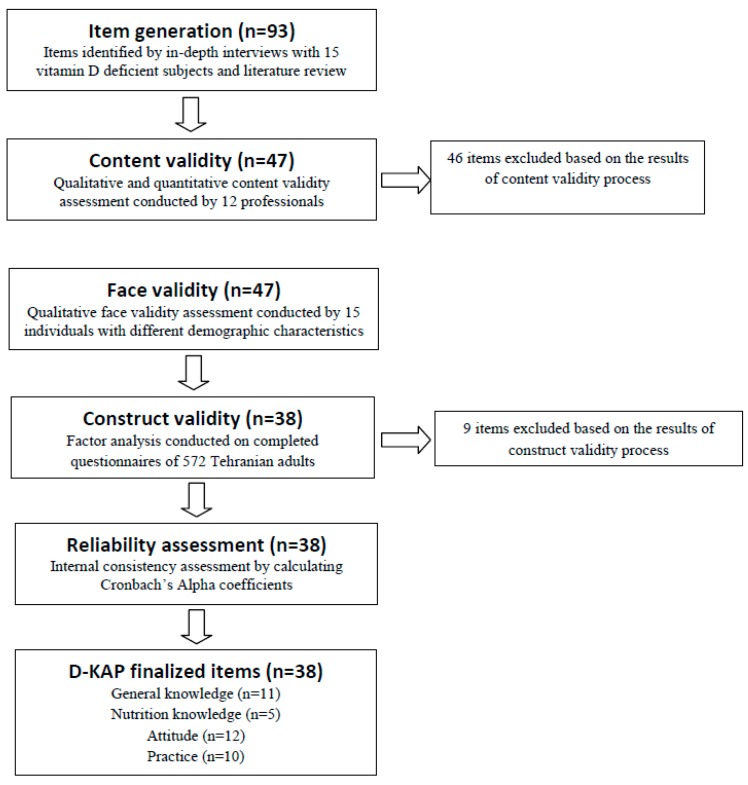
Flowchart of scale development.

Scale development began with item generation and was then followed by different steps assessing face, content, construct validity, and reliability (Figure 1). The final version of D-KAP-38 included 11 general knowledge items, 5 nutrition knowledge items, 12 attitude items, and 10 practice items.

### 2.4. Item Generation

Our item generation was a result of both: (1) In-depth interviews with 15 individuals (5 male and 10 female) belonging to high and low socioeconomic status which were conducted to identify all aspects of the vitamin D-related KAP; (2) An intensive literature review to have a broader view on vitamin D knowledge, attitude, practice. Participants were recruited from endocrine clinics of Tehran, the capital of the Islamic Republic of Iran. The criteria for selection of participants were: (1) Confirmed diagnosis of vitamin D deficiency (25-hydroxy vitamin D < 20 ng/mL) and (2) Participant willingness to share their experiences. The main researcher contacted potential participants to explain the objectives and process of the current research, and if the participants agreed to take part in the research, an interview was scheduled. Overall, fifteen interviews were conducted (mean duration = 30 min) and audio-recorded in a private room using a semi-structured guide consisting of open-ended questions. This enabled respondents to fully explain their conception and experiences regarding the importance of vitamin D and its sources, behaviors related to vitamin D intake, causes of vitamin D deficiency, how to prevent or treat vitamin D deficiency, and sources of information about vitamin D. All interviews recorded were transcribed in Farsi word by word. All items were extracted according to the participant’s responses and related literature available. The final pool of 93 items were categorized into 8 domains: importance of vitamin D deficiency (13 items), prevention and treatment (36 items), attitudinal barriers to sun exposure (16 items), attitudinal barriers to diet containing vitamin D (5 items), attitudinal barriers relating to vitamin D supplements (10 items), behaviors related to sun exposure (7 items), behaviors related to diet (2 items), and behaviors related to vitamin D supplements (4 items).

### 2.5. Validity Assessment

Validity of initial items was assessed through content, face, and construct validity methods. Content validity was confirmed through qualitative and quantitative approaches; to confirm qualitative content validity, a panel of 12 experts in health education, endocrinology, and nutrition completed the questionnaire and were asked to comment on individual items in relation to the accuracy and style. Items were modified, based on the panel comments. Then to confirm quantitative content validity, the content validity ratio (CVR) and content validity index (CVI) were calculated; in this step, the panel were asked to comment independently on the necessity, relevance, clarity, and simplicity of each item. Necessity of items was assessed using a three-point rating scale: (1) not necessary; (2) useful but not essential; and (3) essential. For this purpose a CVR score ranged between −1 (not necessary) and +1 (essential) was computed for each item. The formula of content validity ratio was CVR = (Ne − N/2)/(N/2), in which the Ne is the number of panelists indicating “essential” and N is the total number of panelists. The acceptable CVR values were determined according to the Lawshe scores [22]. The relevance, clarity and simplicity of the items were also assessed by calculating CVI, using a four-point rating scale: (1) not relevant/clear/simple; (2) slightly relevant/clear/simple; (3) relevant/clear/simple; and (4) very relevant/clear/simple. The CVI of each item was determined by the proportion of experts judging the item as relevant/clear/simple (rating 3 or 4). Items with a CVI score lower than 0.70 were eliminated and those items with a CVI between 0.70 and 0.79 were modified according to the recommendations of panel members and research group forums.

Regarding face validity, the D-KAP-38 questionnaire was given to 15 individuals with different age, sex, and educational levels, to ascertain whether the modified items by panelists were relevant to them and, if so, how important each issue was in their daily lives.

To assess construct validity, a total of 572 adults participated in the study. To maximize the heterogeneity of the sample, participants were recruited from public health care centers of two diverse socioeconomic areas of Tehran, one located in the north and the other in the south of Tehran, where the socio economic status of people is mostly high and low, respectively. Exploratory and confirmatory factor analysis (EFA (Explanatory Factorial Analysis) and CFA (Confirmatory Factorial Analysis) were conducted not to reduce items but rather to help place items in the appropriate domains. The maximum length of time for subjects to complete the D-KAP-38 and its demographic form was 20 min.

### 2.6. Reliability Assessment

After performing construct validity and detection of D-KAP-38 subscales, the reliability was assessed, using internal consistency by calculating Cronbach’s Alpha coefficient.

### 2.7. Scoring Method

The possible responses for the items related to the “general knowledge” construct (items 1 to 11) were “Yes/No/I don’t know” which have been scored as 2/0/1 respectively. Hence, the total raw scores of “general knowledge” ranged from 0 to 22 which were proportionately transformed to 0–100. The possible responses for items related to the “nutrition knowledge” construct (items 13 to 17) were “Yes/No/I don’t know” and the scores of 0/2/1 were respectively allocated to all of them except for item 13, which has been inversely scored as 2/0/1. Total raw scores of “nutrition knowledge” ranged from 0 to 10 which have been proportionately transformed to 0–100. The items related to the “attitude” construct (items 20 to 33 except for items 28 and 31) were rated on a 5 Likert scale with the possible responses of “strongly disagree to strongly agree” and the allocated scores of 1 to 5 respectively. Total raw scores of attitude ranged from 12 to 60 and proportionately transformed to 0–100. Finally, the items related to the “practice” construct (items 38 to 47) rated on a 5 Likert scale with the possible responses of “never/rarely/sometimes/often/always”. The scores of 1 to 5 were allocated to the responses of items 38, 39, 40, 41, 43 and 45 and other items were scored inversely. The raw scores of “practice” ranged from 10 to 50 and then proportionately transformed to 0–100. The scoring method for the items remaining in the finalized D-KAP-38 questionnaire is attached as Supplementary Materials.

### 2.8. Statistical Analysis

Range of measurement was based on the percentage of scores at the extremes of the scaling ranges, the maximum (ceiling effect) and the minimum (floor effect) possible scores [23]. Ceiling and floor effects occur when a considerable proportion of subjects score the best/maximum or worst/minimum score, rendering the measure unable to discriminate between subjects at either extreme of the scale. A ceiling or floor effects equal or more than 15%, could suggest a weak detecting power of the applied questionnaire among the target population [24]. Scale internal consistency was determined by calculating Cronbach’s Alpha coefficient, values >0.6 being considered as satisfactory. Exploratory factor analysis was used to assess construct validity and four subscales were extracted. Kaiser–Meyer–Olkin (KMO), Bartlett’s test of sphericity, and total variance explained were used for the evaluation of model adequacy. Principal component extraction method and varimax rotation with Kaiser Normalization was conducted to estimate factor loadings. Factor loadings >0.3 were considered as substantial and items higher than this criterion remained in the constructs. Confirmatory factor analysis with the weighted least squares (WLS) estimation method was performed to test whether the data fit the hypothesized measurement model extracted by EFA. Asymptomatic covariance matrix was applied as a weighted matrix.

Goodness of fit indices and reasonable threshold levels of these indices for CFA were considered as χ^2^/*df* < 3, root mean square error of approximation (RMSEA) and standardized root mean square residual (SRMR) < 0.08, as well as comparative fit index (CFI), goodness of fit index (GFI), normed fit index (NFI), and incremental fit index (IFI) > 0.9 [23].

Modifications of the models in covariance structure were performed based on the largest drop in the overall value of the test statistic to achieve acceptable goodness of fit indices. Conceptual measurement models were tested with and without proposed modifications [19]. Statistical analysis was performed using SPSS 22.0 (SPSS Inc., Chicago, IL, USA) and LISREL 8.80 for windows (Scientific Software International: Lincolnwood, IL, USA, 2006). 

## 3. Results

A total of 572 (54.1% female) adults, aged 30.2 ± 7.9 years (range: 18–68), participated in the study. Socio-demographic status of participants is reported in Table 1. Participants were more likely to be aged < 30 years, married, employed, and living in the north of Tehran. Almost half of the individuals had academic education and vitamin D supplement consumption was rather low (26.1%). Most participants had no children (54.7%).

A satisfactory level of agreement was found among panelists suggesting a good content validity of 47 items of the developed questionnaire (CVR = 0.86 and CVI = 0.89).

Based on face validity results, some items needed to be revised, mostly due to ambiguity, whereas the rest were generally easy to read and understood by subjects. There was no item reduction in this step.

In case of EFA, Kaiser-Meyer-Olkin (KMO) showed a reasonable fit of the model (KMO = 0.658), and the Bartlet’s test (χ^2^ = 2762.9, *df* = 1081, *p* < 0.001) confirmed the sphericity assumption. The factor analysis identified four factors with eigen values 2, factors which made intuitive sense and were characterized as follows: general knowledge, nutrition knowledge, attitude, and practice. The final analysis was repeated with these four factors using a varimax rotation.

Table 2 presents the 47 items included in the factor analysis with their associated factor loadings. Among 47 items, 7 items (q12, q18, q19, q34, q35, q36 and q37) had factor loadings less than 0.3 and 2 items (q28 and q31) were not consistent with their related factors. Overall, the total percentage of variance was 25.57 and percentage of variance explained by general knowledge, nutrition knowledge, attitude, and practice, was 6.99, 5.15, 6.99, and 6.81, respectively.

Based on 47 items which have been loaded in four explored factors, EFA was conducted on a random sample of 50% of subjects (285 cases as “exploring” data) and then CFA was used on the remaining sample (287 cases as “testing” data). The second hypothesized CFA model, with 38 remaining items in four constructs, had acceptable goodness of fit indices (Table 3).

**Figure 2:**
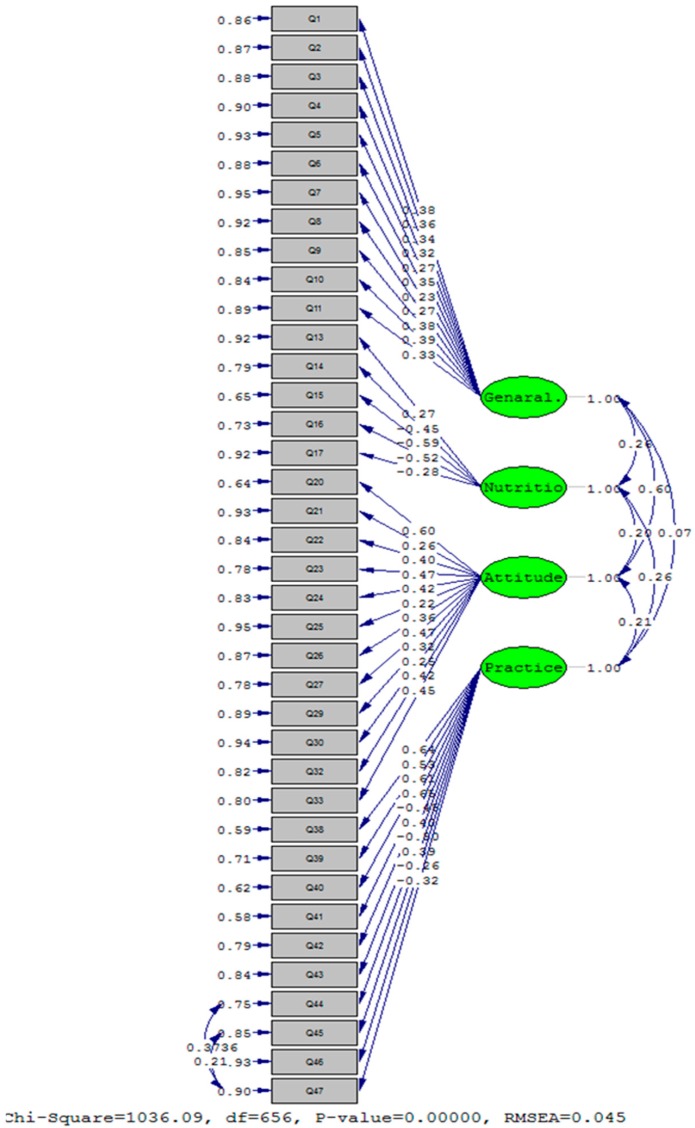
Standardized factor loadings of measurement model of D-KAP-38. Four latent constructs and 38 observed items were included in the confirmatory factor analysis (CFA) model. Based on the results of chi-square statistic and in order to achieve acceptable fit indices, the correlation of “q44, q47” = 0.37, “q45, q47” = 0.21, and “q45, q46” = −0.36 have been added to model.

Figure 2 indicates the conceptual framework of CFA model with four constructs and 38 finalized items. Standardized factor loadings are displayed above pathways. Also, correlation between constructs and its significance criteria (T-values) was found to be significant on the two sided pathways.

For the “general knowledge” construct, the minimum and maximum loadings are related to q7 (λ = 0.23, T = 2.22) and q10 (λ = 0.39, T = 4.78), respectively. For the “nutrition knowledge” construct the minimum and maximum loadings are related to q13 (λ = 0.27, T = 2.62) and q15 (λ = −0.59, T = −5.52), respectively. For the “attitude” construct the minimum and maximum loadings are related to q25 (λ = 0.22, T = 5.98) and q20 (λ = 0.60, T = 11.10), respectively, and for the “practice” construct the minimum and maximum loadings are related to q46 (λ = −0.26, T = −8.88) and q41 (λ = 0.65, T = 22.22), respectively.

The mean ± SD subscale scores and number of items in each subscale are presented in Table 4; no ceiling effects were observed except for general knowledge (1.2%). Floor effects detected were 0%, 1.1%, 2.4%, and 8.7% for practice, attitude, general knowledge, and nutrition knowledge, respectively. General knowledge had the highest score (79.59 ± 14.52) and nutrition knowledge had the lowest (42.58 ± 20.40) among the four sub-scales. The Cronbach’s alpha coefficients were calculated for each subscale and ranged between 0.60 and 0.74.

**Figure 3:**
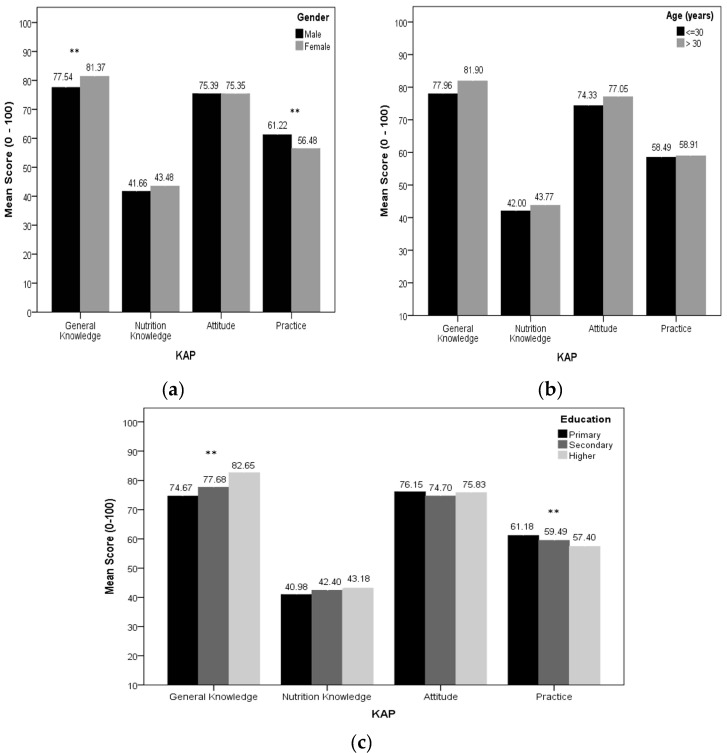
Mean comparison of D-KAP-38 sub-scales for sex (**a**); age (**b**); and education (**c**) groups (** *p* < 0.01).

The general knowledge score was higher among females than males; however, their practice score was lower. Furthermore, general knowledge and attitude score was higher among subjects aged >30 years compared to those aged ≤30 years. The general knowledge score was higher among subjects with higher education, although their practice score was lower (Figure 3).

## 4. Discussion

This study is one of the first efforts to develop and assess the psychometric properties of a KAP questionnaire regarding vitamin D among an urban Eastern-Mediterranean population. Our results support the initial reliability and validity of this developed questionnaire. In this study, 25 questionnaires (4.4%) had more than 15% missing items and were excluded from the analysis. This low percentage of missing values showed the acceptable feasibility of the questionnaire. Internal consistency of different constructs showed that Cronbach’s alpha generally exceeded the standard of >0.60, confirming the reliability of D-KAP-38.

In the case of quantitative content validity, a satisfactory level of agreement (CVI = 0.89 and CVR = 0.86) was found among panelists, suggesting that the scale had a good content validity. Despite the substantial theory of KAP which considers three constructs, i.e., knowledge, attitude and practice in the developed questionnaire, the current results of exploratory factor analysis (EFA) suggested a four-factor structure as an optimized structure that emerged from this item pool. In this regard, the knowledge construct was divided into two factors: (1) Nutrition knowledge, which contained questions related to nutrition and (2) General knowledge which encompassed non nutritional vitamin D-related questions, including the importance of vitamin D, sun exposure and using supplements. This four factor structure, confirmed by confirmatory factor analysis (CFA), indicates the acceptable fit of the proposed models.

The above mentioned four factor structure of D-KAP-38, allowed us to conduct a deeper analysis of vitamin D-related knowledge in the population studied. Based on the current results, despite participants having an acceptable general knowledge regarding vitamin D, there was a lack of nutritional awareness regarding vitamin D in both men and women, findings in agreement with the results of other studies that reported poor awareness on nutritional sources of vitamin D in general populations [13,15,24]. Toher et al. revealed that almost 20% of their study population had no knowledge of any nutritional sources of vitamin D, and among those who did, there was apparent confusion about the best sources of this vitamin [25]; thus, it seems considering these results, assessing individuals’ general and nutritional vitamin D-related knowledge separately is essential and definitely beneficial in planning health promotion programs.

Our results showed an acceptable known group validity of D-KAP-38. In this study, as hypothesized, women had significantly higher general vitamin-D-related knowledge and lower practice, compared to men. Consistent with our findings, previous articles had a marked lack of knowledge in their study populations [17,26]. Higher awareness regarding vitamin D in women was mainly attributed to their desire to obtain health information from media and professionals. However, poorer practices in women could be a result of several socio-environmental barriers, limiting women from sun exposure and also their desire to use sunscreen.

The current results showed significantly higher scores in general knowledge and attitude among individuals aged ≥30 years, in agreement with findings of an earlier study by Kung et al.; several personal and environmental factors influence this relationship, e.g., middle-aged people, especially women, are advised to take calcium and vitamin D supplements to prevent osteoporosis and its complications [13,27]. Although it seems that middle-aged individuals usually get more medical attention and also are more interested in media, which could have improved their self-care, more investigations are needed for results to be confirmed. Furthermore, it is reasonable to assume different vitamin D-related KAP scores in people with different educational levels. Based on our results, compared to those with primary and secondary education, higher educated individuals have significantly higher knowledge and poorer practices, similar to a study from Kuwait, which also reported higher levels of vitamin D-related awareness in educated people [15]; this conflict between knowledge and behavior among educated people may be a result of educated people spending more time indoors and having less leisure time for sun exposure.

The main strength of this study was development and evaluation of the psychometric properties of a vitamin D-related questionnaire for the first time. Furthermore, we considered a holistic approach to all aspects of the vitamin D-related KAP. Although the current questionnaire has been developed in Iran, it would be usable in other countries with similar socio-economic status and cultural backgrounds. In addition, it could be tailored to communities with different socio-cultural aspects after the required modifications. However, a number of limitations should also be considered: first, individuals were selected from only one city of Iran and further investigations of other Iranian populations are recommended. Furthermore, due to unavailability of the participants for the following 2-week assessment we were unable to conduct test re-test and confirm reproducibility of the questionnaire.

## 5. Conclusions

In conclusion, our results confirm the initial validity and reliability of the D-KAP-38. Further investigations in different urban and rural populations are recommended. In addition, further translational studies are needed to investigate the correlation between 25-hydroxy vitamin D and D-KAP-38 scores to determine contributions of vitamin D-related knowledge, attitude and practices to the vitamin D status of individuals. This information would be valuable in designing and implementing public health programs improving vitamin D levels in general populations. Finally, future studies would be required to develop a shorter form of D-KAP-38 with reasonable sensitivity for detecting overall vitamin D-related KAP; this would be more convenient and user-friendly for use in community-based health promotion programs.

## Figures and Tables

**Table 1:** Baseline characteristics of the participants (*n* = 572).

Variable	Number	%
Female	308	54.1
Married	397	70.0
Age (years)		
≤30	334	61.9
>30	206	38.1
Parity		
0	313	54.7
1	127	22.2
≥2	132	23.1
Education level		
Primary	74	13.0
Secondary	222	39.0
Higher	273	48.0
Employed	324	58.1
Residential area		
North of Tehran	184	36.0
South of Tehran	327	64.0
Intake of vitamin D supplement	148	26.1

**Table 2:** Factor loading matrix of D-KAP-38.

*N*	Questions	Factor Loading			
		General Knowledge	Nutrition Knowledge	Attitude	Practice
1	People, who work indoors, are at high risk of vitamin D deficiency.	**0.642**	−0.156	0.004	0.059
2	Vitamin D intake more than dietary recommendations could be harmful.	**0.599**	−0.108	−0.130	−0.043
3	Elderly people are at high risk of vitamin D deficiency.	**0.498**	0.149	0.037	0.102
4	Inappropriate dietary intakes are related to vitamin D deficiency.	**0.495**	0.280	−0.074	−0.083
5	Vitamin D supplement intake requirements, differ for different age groups.	**0.449**	0.263	0.110	−0.092
6	Pregnant and lactating women are at high risk of vitamin D deficiency.	**0.439**	−0.012	0.048	0.040
7	Most of the vitamin D required is produced when the skin is directly exposed to the sun.	**0.395**	−0.171	0.081	−0.069
8	Currently, vitamin D deficiency is one of the most important health issues in Iran.	**0.379**	0.265	0.227	0.161
9	Bone pain and fatigue are among the vitamin D deficiency symptoms.	**0.330**	0.227	−0.040	0.133
10	Vitamin D supplement intake requirements, differ in various seasons of the year.	**0.321**	0.127	0.202	−0.124
11	Both men and women are at risk of vitamin D deficiency.	**0.320**	0.068	−0.106	0.211
12	All age groups are at risk of vitamin D deficiency.	0.249	0.153	0.087	−0.057
13	Fatty fishes are one of the main dietary sources of vitamin D.	**0.394**	**0.336**	0.097	−0.042
14	Dairy products are one of the main dietary sources of vitamin D.	−0.125	**−0.646**	−0.051	−0.100
15	Eggs are one of the main dietary sources of vitamin D.	−0.133	**−0.571**	−0.171	−0.046
16	Meat and poultry are the main dietary sources of vitamin D.	0.029	**−0.526**	−0.074	0.044
17	Fruits are one of the main dietary sources of vitamin D.	0.053	**−0.300**	0.051	−0.052
18	Clothes prevent the skin from producing vitamin D.	0.125	0.159	0.054	0.072
19	Sun exposure through glass, prevents the skin from producing vitamin D.	0.048	0.154	0.031	0.023
20	Urbanization prevents sun exposure and production of required vitamin D.	0.212	−0.039	**0.575**	−0.261
21	A shortage of public places for outdoor activities prevents the sun exposure required for production of vitamin D.	0.235	−0.151	**0.558**	0.005
22	Full time indoor occupation prevents the sun exposure required for production of vitamin D.	**0.300**	−0.131	**0.552**	−0.089
23	Inefficient education regarding benefits of sun exposure prevents production of required vitamin D through sun exposure.	**0.323**	−0.061	**0.531**	0.070
24	The undesirable taste of sea foods for Iranians is one of the barriers to their consumption of dietary sources of vitamin D.	0.257	0.140	**0.467**	0.019
25	In vitamin D deficiency, supplement intake is more effective compared to dietary intake and sun exposure.	−0.182	0.075	**0.464**	0.218
26	Taking vitamin D supplement, unless recommended by physicians is wrong.	0.036	−0.080	**0.430**	0.019
27	Unwillingness of individuals to take vitamin D supplement is one of the barriers of providing this nutrient.	0.020	0.161	**0.424**	0.192
28	Taking supplements is beneficial in case of not consuming dietary sources of vitamin D.	0.081	0.041	**−0.401**	−0.116
29	Taking supplements is necessary for treatment of vitamin D deficiency but not for its prevention.	−0.155	0.135	**0.398**	−0.008
30	Permanent using of sunscreens on face, neck and hands prevents the sun exposure required for production of vitamin D.	−0.078	0.026	**0.396**	0.077
31	To prevent vitamin D deficiency, taking supplements is easier, compared to dietary intakes and sun exposure.	−0.255	0.217	**0.355**	0.103
32	Taking supplements is only necessary in case of lack of exposure to sunlight.	0.165	0.258	**0.301**	0.057
33	A high expense of dietary sources of vitamin D is one of the barriers of providing this nutrient.	0.009	−0.325	**0.303**	0.257
34	Cloudy weather prevents absorption of ultra violet and producing vitamin D by skin.	−0.018	0.028	0.251	0.006
35	Vitamin D supplementation is recommended for individuals suffering from vitamin D deficiency.	0.026	0.113	0.224	−0.042
36	Vegetarians are at high risk of vitamin D deficiency.	0.112	0.128	0.204	0.130
37	Air pollution prevents absorption of ultra violet and production of vitamin D by skin.	0.130	0.152	0.169	0.080
38	For sufficient exposure to sunlight I regularly engage in outdoor physical activities.	−0.073	0.038	0.089	**0.691**
39	To be vitamin D sufficient, I consume fortified milk.	−0.063	0.102	0.119	**0.661**
40	In order to be vitamin D sufficient, I consume fish at least twice a week.	−0.032	0.170	0.125	**0.618**
41	For sufficient exposure to sunlight I walk outdoors daily.	0.096	−0.055	0.055	**0.616**
42	I use caps/hats to avoid severe sun exposure.	−0.122	0.078	0.038	**−0.515**
43	To be vitamin D sufficient, I take vitamin D supplements.	−0.058	0.242	0.161	**0.512**
44	I use sunscreen on my hands.	0.069	−0.178	0.032	**−0.455**
45	During the day I am directly exposed to sunlight (outdoors).	0.028	−0.263	0.015	**0.395**
46	During the day I am indirectly exposed to sunlight (through glass).	−0.071	0.265	−0.126	**−0.316**
47	I use sunscreen on my face.	−0.072	−0.265	0.056	**−0.300**

**Table 3:** Fit indices based on 38 items for measurement model of D-KAP-38.

	χ^2^	DF	χ^2^/*df*	RMSEA	GFI	CFI	SRMSR	NFI	IFI
Model ^1^	1177.55	659	1.78	0.05	0.90	0.99	0.07	0.99	0.99
Model ^2^	1036.10	656	1.58	0.05	0.92	0.99	0.07	0.99	0.99

χ^2^: Chi-Square value; DF: Degrees of Freedom; RMSEA: Root Mean Square Error of Approximation; GFI: Goodness of Fit Index; CFI: Comparative Fit Index; SRMSR: Standardized Root Mean Square Residual; NFI: Normed Fit Index; IFI: Incremental Fit Index. ^1^ Unmodified model; ^2^ Modified by adding covariances between items 44 and 47, items 45 and 47, and items 45 and 46.

**Table 4:** Mean, standard deviation, percentage of floor and ceiling effects, and Cronbach’s α for vitamin D-related knowledge, attitude and practices of study participants (*n* = 572).

Constructs	*n*	Mean	Median	SD	Min	Max	Floor (%), Ceiling (%)	Cronbach’s α
General knowledge	11	79.6	81.8	14.5	4.5	100.0	2.4, 1.2	0.62
Nutrition knowledge	5	42.6	40.0	20.4	0.0	100.0	8.7, 0.00	0.60
Attitude	12	75.4	75.0	9.5	50.9	100.0	1.1, 0.00	0.68
Practice	10	58.7	58.0	8.6	32.0	94.0	0.00, 0.00	0.74

## Supplementary Materials

The following are available online at www.mdpi.com/2072-6643/9/5/471/s1 D-KAP-38 questionnaire; the scoring instructions of D-KAP-38.
