# Supplementary material for: Psychometric Properties of a Developed Questionnaire to Assess Knowledge, Attitude and Practice Regarding Vitamin D (D-KAP-38)
Source: Nutrients. 2017 May 8;9(5):471. doi: 10.3390/nu9050471 (PMC5452201; doi:10.3390/nu9050471)
Supplement: Supplementary file 1 [file nutrients-09-00471-s001.pdf]

Supplementary Materials

Knowledge, attitude and practice (KAP) regarding vitamin D

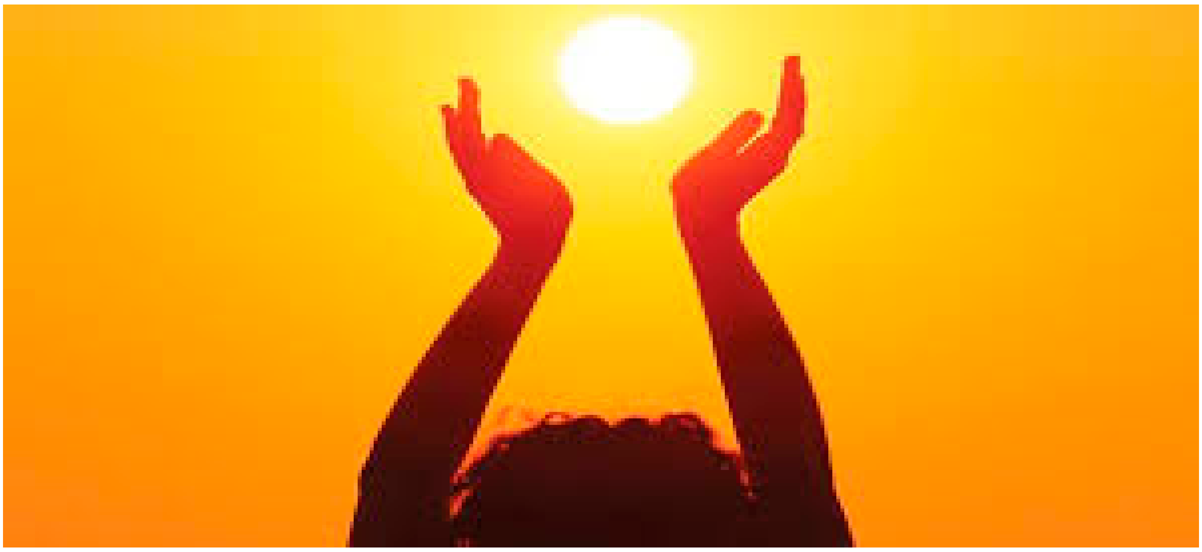

D-KAP-38 questionnaire

Guidance note

- ✓ On the following page, there is a list of questions regarding vitamin D nutrition.
- ✓ The questionnaire consisting of four sections: 1) general Knowledge; 2) nutrition knowledge; 3) attitude; and 4) practice.
- ✓ Please provide the best answer to each question and leave no blanks; if you have a question please ask for help.
- ✓ Each question includes the answer option 'No idea' or 'I don't know'. This should be checked if a respondent does not know the answer or does not provide an answer to a

	General knowledge	Yes	No	I don’t know
1	People, who work indoors, are at high risk of vitamin D deficiency.			
2	Vitamin D intake more than dietary recommendations could be harmful.			
3	Elderly people are at high risk of vitamin D deficiency.			
4	Inappropriate dietary intakes are related to vitamin D deficiency.			
5	Vitamin D supplement intake requirements, differ for different age groups.			
6	Pregnant and lactating women are at high risk of vitamin D deficiency.			
7	Most of the vitamin D required is produced when the skin is directly exposed to the sun.			
8	Currently, vitamin D deficiency is one of the most important health issues in our country.			
9	Bone pain and fatigue are among the vitamin D deficiency symptoms.			
10	Vitamin D supplement intake requirements, differ in various seasons of the year.			
11	Both men and women are at risk of vitamin D deficiency.			
	**Nutrition knowledge**	Yes	No	I don’t know
12	Fatty fishes are one of the main dietary sources of vitamin D.			
13	Dairy products are one of the main dietary sources of vitamin D.			
14	Eggs are one of the main dietary sources of vitamin D.			
15	Meat and poultry are the main dietary sources of vitamin D.			
16	Fruits are one of the main dietary sources of vitamin D.			

What do you think of each questions based on your previous information and activities.

	Attitude	Strongly agree	Agree	No idea	Disagree	Strongly disagree
17	Urbanization prevents sun exposure and production of required vitamin D.					
18	A shortage of public places for outdoor activities prevents the sun exposure required for production of vitamin D.					
19	Full time indoor occupation prevents the sun exposure required for production of vitamin D.					
20	Inefficient education regarding benefits of sun exposure prevents production of required vitamin D through sun exposure.					
21	The undesirable taste of sea foods for Iranians is one of the barriers to their consumption of dietary sources of vitamin D.					
22	In vitamin D deficiency, supplement intake is more effective compared to dietary intake and sun exposure.					
23	Taking vitamin D supplement, unless recommended by physicians is wrong.					
24	Unwillingness of individuals to take vitamin D supplement is one of the barriers of providing this nutrient.					
25	Taking supplements is necessary for treatment of vitamin D deficiency but not for its prevention.					
26	Permanent using of sunscreens on face, neck and hands prevents the sun exposure required for production of vitamin D.					
27	Taking supplement is only necessary in case of lack of exposure to sunlight.					
28	A high expense of dietary sources of vitamin D is one of the barriers of providing this nutrient.					

In past 3 months what is your behavior toward following questions?						
	**Practice**	Always	Often	Sometimes	Rarely	Never
29	For sufficient exposure to sunlight I regularly engage in outdoor physical activities.					
30	To be vitamin D sufficient, I consume fortified milk.					
31	In order to be vitamin D sufficient, I consume fish at least twice a week.					
32	For sufficient exposure to sunlight I walk outdoors daily.					
**33**	I use caps/hats to avoid severe sun exposure.					
**34**	To be vitamin D sufficient, I take vitamin D supplements.					
**35**	I use sunscreen on my hands.					
**36**	During the day I am directly exposed to sunlight (outdoors).					
**37**	During the day I am indirectly exposed to sunlight (through glass).					
**38**	I use sunscreen on my face.					

The scoring instructions of D-KAP-38

General knowledge (Q1-Q11): The possible responses are “Yes/No/I don’t know” which should be scored as 2/0/1 respectively. Hence, the total raw scores of “general knowledge” ranged from 0 to 22 which will be proportionately transformed to 0-100.

Nutrition Knowledge (Q12-Q16): The possible responses are “Yes/No/I don’t know” and the scores of 0/2/1 are respectively allocated to all of them except for Q12, which should be inversely scored as 2/0/1. Total raw scores of “nutrition knowledge” ranged from 0 to 10 which will be proportionately transformed to 0-100.

Attitude (Q17-Q28): The related questions are rated on a 5 Likert scale with the possible responses of “strongly disagree to strongly agree” and the allocated scores of 1 to 5 respectively. Total raw scores of attitude range from 12 to 60 and proportionately transform to 0-100.

Practice (Q29-Q38): The related questions are rated on a 5 Likert scale with the possible responses of “never/rarely/sometimes/often/always”. The scores of 1 to 5 will be allocated to the responses of questions 29, 30, 31, 32, 34 and 36 and other questions should be scored inversely. The raw scores of “practice” ranged from 10 to 50 and then proportionately will be transformed to 0-100.
